# Plant-Derived Antioxidants Protect the Nervous System From Aging by Inhibiting Oxidative Stress

**DOI:** 10.3389/fnagi.2020.00209

**Published:** 2020-07-14

**Authors:** Xiaoji Cui, Qinlu Lin, Ying Liang

**Affiliations:** Molecular Nutrition Branch, National Engineering Laboratory for Rice and By-product Deep Processing, College of Food Science and Engineering, Central South University of Forestry and Technology, Changsha, China

**Keywords:** plant-derived antioxidants, nerve aging, neuroprotection, nerve cells, nervous system

## Abstract

Alzheimer’s disease (AD) has become a major disease contributing to human death and is thought to be closely related to the aging process. The rich antioxidant substances in plants have been shown to play a role in delaying aging, and in recent years, significant research has focused on also examining their potential role in AD onset and progression. Many plant-derived antioxidant research studies have provided insights for the future treatment and prevention of AD. This article reviews various types of plant-derived antioxidants with anti-aging effects on neurons. Also it distinguishes the different types of active substances that exhibit different degrees of protection for the nervous system and summarizes the mechanism thereof. Plant-derived antioxidants with neuroprotective functions can protect various components of the nervous system in a variety of ways and can have a positive impact on interventions to prevent and alleviate AD. Furthermore, when considering neuroprotective agents, glial cells also contribute to the defense of the nervous system and should not be ignored.

## Introduction

Plants have developed a wide variety of active substances, including many antioxidants, to adapt to their constantly evolving environment. Accumulating evidence shows that some antioxidants in plants can effectively scavenge free radicals, protect cells, delay aging, and prevent diseases related to aging (Hassan et al., 2017). At present, three major diseases, including cancer, cardiovascular disease, and Alzheimer’s disease (AD), are closely related to the aging of individuals. In particular, AD is closely related to the pathological changes of nerve cells that manifest and persist across the human lifetime. Such neuronal changes, or lesions, are characterized by tangles of nerve fibers and the increased presence of β-amyloid and hyperphosphorylated tau in the brain, which ultimately leads to a decline in nervous system function. Since there is no effective treatment for AD in modern medicine, and since the prevalence of AD is positively correlated with an individual’s age, the risk of AD can be effectively reduced by delaying or mitigating the negative effects on the nervous system that are associated with the aging process (Goedert, 2015). As the main source of antioxidants in humans, plants play an important role in the anti-aging process of the human body. At present, the use of plant-derived antioxidants to reduce the damage caused by oxidative stress to nerve cells has been well documented. Accordingly, various types of nerve cells strategically coordinate functions to contribute to the collective well-being of the nervous system. Therefore, the function of plant antioxidants applies not only to protecting nerve cells but also to protecting the entire nervous system of the body. This article reviews the role of various antioxidants on AD progression and describes the mechanistic role of such substances on the nervous system.

## The Protective Effect of Plant-Derived Antioxidants on Nerve Cells

**FIGURE 1:**
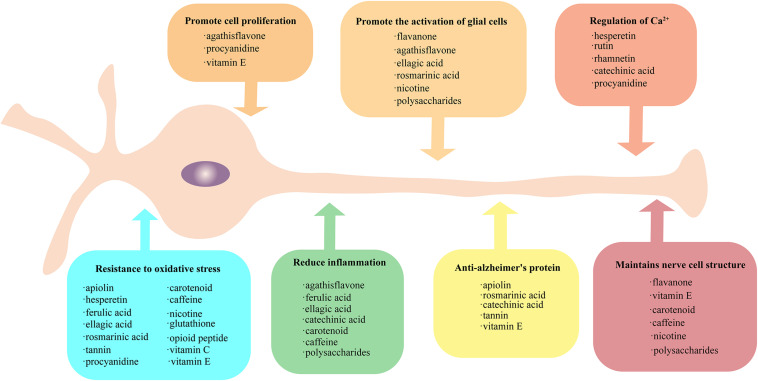
Protective method of plant-derived active substances on nerves. Active plant substances exhibit rich neuroprotective methods, which can be grouped into seven categories: (1) promote the activation of glial cells, (2) promote cell proliferation, (3) regulation of Ca^2+^, (4) maintain nerve cell structure, (5) provide resistance to oxidative stress, (6) reduce inflammation, and (7) anti-Alzheimer’s protein. The active substances falling into each of these seven categories are provide in the respective boxes. All the active substances could resist oxidative stress, and some substances have similar protection patterns.

Plants contain a wide variety of antioxidants that resist various naturally occurring environmental threats and which are metabolized primarily during aerobic activities. Free radicals become oxidized when they combine with oxygen-containing substances and can damage the host organism by stealing surrounding electrons (Wojtunik-Kulesza et al., 2016). Antioxidants in plants can prevent the damage caused by free radicals (Liochev, 2013) by preventing electron transfer. Free radicals in the human body that are not cleared fast enough can damage cells and eventually lead to local apoptosis and in turn global aging of the body. For cells in the nervous system, degenerative diseases that affect neurons and nerve bundles are related to the damage caused by oxidative free radicals. The ability of plant-derived antioxidants to scavenge free radicals can reduce the damage of nerve cells caused by oxidative stress and help maintain a more active physiological state. In addition, plant-derived antioxidant substances have a wide range of cell types that aid in the removal of nerve cell free radicals and can effectively protect most of the nervous system. Antioxidants such as polyphenols, vitamins, alkaloids, polysaccharides, and active peptides (Figure 1) help to maintain the structure and function of neurons and prolong their healthy state (Del Rio, 2015). In subsequent sections, we will briefly review each of these substances and how they protect the brain and nerve cells against the damaging effects of aging.

### Polyphenols

Polyphenols are the most abundant antioxidant in plants and have an excellent ability to capture oxidative free radicals (Jiang et al., 2016). Polyphenols can be sub-classified into flavonoids, phenolic acids, and tannins depending on their physical structure.

#### Flavonoids

Flavonoids can be isolated from vegetables, fruits, and rice. This class of polyphenols have a lower redox potential than oxygen and superoxide radicals, making these antioxidants better suited to bind active oxygen (Duthie and Crozier, 2000). Recent studies have shown that flavonoids can be absorbed into the blood and tissue through the intestine and can easily reach the brain through the blood–brain barrier. Once in the brain, flavonoids and their metabolites protect the cells inside from oxidative stress (Pietta, 2000). These flavonoids can also induce glial cell secretion of nerve growth factor to prevent the degradation of dopaminergic neurons in the substantia nigra (Jaeger et al., 2018). In an exemplary study, Zhao et al. (2013) found that one specific flavonoid called apigenin protects the extracellular environment in the nervous system (Jung and Kim, 2018) by scavenging free radicals through the release of anti-amyloid proteins. This study also found that flavonoids can protect neurons from axonal degradation, myelin rupture (Zhao et al., 2013), *trans-*differentiation, and Schwann cell proliferation (“Correction: Curcumin and Apigenin – novel and promising therapeutics against chronic neuroinflammation in Alzheimer’s disease,” No author, 2015) through the Krox20 pathway and extracellular signal-regulated kinase-independent processes. In another experiment, the hesperidin flavonoid activated the secretion of superoxide dismutase and glutathione (Jeong et al., 2019) in the mouse brain. Hesperidin (Matias et al., 2017), rutin (El-Sayed el et al., 2008), and rhamno (Omar et al., 2017) can reduce the amount of free Ca^2+^ located between brain cells when fighting against neurogenic excitotoxicity and can attenuate the decrease of the mitochondrial membrane potential and increase caspase-3 activity (Lutz et al., 2015). In addition, recent studies have shown that treating neurons in the cerebral cortex with microglial cells cultured with agathisflavone may protect them from oxidative stress by modulating estrogen signaling. Agathisflavone has been found to significantly increase the number of neuronal progenitor cells and mature neurons without increasing astrocytes or microglia, and also to effectively reduce the inflammatory factors factor-α, interleukin-6, interleukin-1β, NO, and PGE2 (Bakhtiari et al., 2017). Other studies have shown that agathisflavone, which regulates estrogen signaling, stimulates neuronal production *in vitro* and enhances the neuroprotective properties of microglia and astrocytes.

#### Phenolic Acids

Phenolic acid refers to a compound having several phenolic hydroxyl groups on the same benzene ring and is an allelochemical substance that regulates the growth state of plants through the process of allelopathy. The antioxidant activity of phenolic acids in organisms is easily affected by environmental influences and often includes an oxidative effect under the action of Cu^2+^.

Ferulic acid is a ubiquitous phenolic acid found in the leaves and seeds of plants. The conjugated structure of the ferulic acid molecule can form a relatively stable phenoxy radical in ultraviolet light to terminate the free radical chain reaction (Dos Santos Souza et al., 2018). Ferulic acid protects neurons from oxidative stress by inhibiting the activation of p38 MAPK, caspase-3, and COX-2 (Graf, 1992), and by downregulating damage caused by the MEK/ERK/p90RSK signaling pathway (Lin et al., 2015). In mice, it was found that ferulic acid can increase SOD, CAT, and GSH-Px activity in the brains of depressed mice and reduce TBA-RS levels in the blood, hippocampus, and cerebral cortex (Lin et al., 2015). Additionally, ferulic acid can significantly reduce seizure intensity, myoclonic spasms, and cognitive decline in epileptic mice (Lenzi et al., 2015), and can reduce the Bax/Bcl2 ratio in dopaminergic neurons in the striatum in mice with Parkinson’s disease (PD) (Hassanzadeh et al., 2017). Furthermore, ferulic acid treatment in a mouse model of neuroinhibition reduces p-JNK, p-NFκB in glial cells of the mouse hippocampus and activates mitochondrial apoptotic molecules (Bax, cytochrome c, caspase-3, and PARP-1) in small/medium glial cells to induce anti-inflammatory effects by interfering with the TLR4/MD2 complex binding site (Nagarajan et al., 2015).

Another study using ellagic acid to treat oxygen-glucose deprivation and reoxygenation models of rat cortical neurons cultured *in vitro* found a significantly reduced volume of cerebral infarction and improved neurological deficit score in rats by increasing the Bcl-2/Bax ratio (Rehman et al., 2019). Similarly, Wang et al. (2019) found that the antioxidant defense of ellagic acid can cause astrocyte proliferation, glial cell line-derived neurotrophic factor (GDNF) release, and Nrf2 activation (Liu et al., 2017). Ellagic acid has also been shown to increase the activity of GSH-Px and SOD and to decrease the level of MDA in the striatum of PD mice (Wang et al., 2019). Even more, ellagic acid has additionally been shown to increase monoamine oxidase B (MAO-B), nuclear factor (erythrocyte derivation 2), Nrf2, and heme oxygenase 1 (HO-1) and reduce the loss of tyrosine hydroxylase (TH)-positive neurons in the substantia nigra pars compacta (SNC) (Sarkaki et al., 2016). In a rat model of arsenic-induced injury, the hippocampus was treated with ellagic acid, which was shown to regulate total ROS production, apoptosis markers, BAX and Bcl2, and inflammatory markers IL-1β, TNFα, and INFγ. At the same time, ellagic acid also prevented the decrease of the mitochondrial membrane potential (Baluchnejadmojarad et al., 2017).

Yet another phenolic acid, rosmarinic acid, has been found to reduce the amount of reactive oxygen species and malondialdehyde, which in turn attenuates cellular oxidative stress and protects hydrogen peroxide-treated glial cells (Firdaus et al., 2018) by phosphorylating protein kinase B (Akt), Ser9 Glycogen synthase kinase-3β (GSK-3β), and Fyn. Rosmarinic acid further regulates the activity of Nrf2 to protect PC-12 cells from oxidative stress (Gao et al., 2005) induced by amyloid β (Aβ). This substance can also reduce the oxidative stress (Lee et al., 2008) on SH-SY5Y (human dopaminergic neuron cells) by controlling the upregulation of Bax and the downregulation of Bcl-2, and reduce mitochondrial membrane potential by reducing reactive oxygen species. At the same time, rosmarinic acid promotes the upregulation of TH and brain-derived neurotrophic factor (BDNF) genes to alleviate the cytotoxicity of H_2_O_2_-induced N2A cells (Ghaffari et al., 2014). After cerebral ischemia/reperfusion in rats, rosmarinic acid was found to increase the phosphorylation of Akt1, decrease the phosphorylation of JK3, and decrease the expression of cleaved caspase-3 to protect hippocampal neurons in the brain (Zhang et al., 2017c). A rat AD model was established by injecting Aβ4 into the bilateral lateral ventricle of rats, which resulted in a decrease in acetylcholine content and acetylcholinesterase activity. Treatment with rosmarinic acid prevented the observed changes in the Aβ group and in another study attenuated Aβ staining and astrocyte activation and enhanced auditory abilities (Kantar Gok et al., 2018).

#### Tannins

Tannins are widely distributed in plants and usually refer to plant polyphenols with a relative molecular mass of 500–3000 u. The antioxidant ability of tannins is related to the position and binding mode of the phenolic hydroxyl groups. Nevertheless, the tannin-like antioxidant ability is significantly different across different sub-structures.

Catechin and its derivatives are the main functional components in tea and many studies have shown that the effects of catechin on mitochondria-related pathways can prevent neurodegeneration and delay the decline of brain function (Assuncao and Andrade, 2015). Catechin protects Aβ- and 6-OHDA-induced neuronal apoptosis by activating the protein kinase C (PKC) pathway and PI3K/AKT, which inhibits the MAPKs pathway (Chen et al., 2018). Specifically, epigallocatechin-3-gallate (EGCG) was found to enhance the clearance of AD-associated phosphorylated tau species in neurons (Chesser et al., 2016) and to improve cell metabolism and reduce oxidative stress to protect motor neurons (Che et al., 2017). The use of EGCG and related phenolic compounds can also redirect the amyloid-forming aggregation pathway of transgenic *Caenorhabditis elegans* strains that express amplified ATX3 (amyloid) toward non-toxic aggregation and prevent calcium influx-mediated cytotoxicity of nerve cells (Visentin et al., 2017). When DOX has been used to induce memory deficits in Wistar rats, catechin treatment was able to reduce neuronal toxicity and improve cognitive performance in a time- and dose-dependent manner (Cheruku et al., 2018).

Tannin is the main enzyme involved in the production and deposition of Aβ peptide, which regulates β-secretase (BACE1) activity and is a natural inhibitor of protein expression. Tannin also destabilizes neurotoxic Aβ fibrils and inhibits *in vitro* aggregation of tau peptides (Braidy et al., 2017) and can also reduce MDA-enhanced SOD activity and higher respiratory factor-1 (NRF-1) in ischemic rats.

Grapes and lotus roots contain a wide range of tannins, the main functional component of which is proanthocyanidin (PC). Song et al. (2019) first confirmed that treatment of glutamate with PCs induced HT22 cells to block the phosphorylation of MAPK, including ERK1/2 and p38. Lotus anthocyanins can also regulate the activation of the Bcl-2 and Bcl-xl proteins, the latter of which protects hippocampal neurons from damage in extremely low-frequency electromagnetic fields and increases in Ca^2+^ levels. Damage to mice by extremely low-frequency electromagnetic fields revealed that lotus root PCs can mediate calcium signals and double the activity of messenger systems through Ca^2+^, CaMK II/CREB, BDNF, and the DG/PKC/MAPK signaling pathways to reverse mouse hippocampal cell oxidative stress and BDNF levels. Additionally, behavioral changes in the 6-OHDA mouse model were reversed by the PI3K/Akt signaling pathway (Zhang et al., 2017a), and the reduced number of dopaminergic cells and the levels of dopamine and its metabolites DOPAC and HVA were restored (Datla et al., 2007) when treated with PCs.

### Vitamins

Vitamins in the body have always been regarded as one of the most bioavailable nutrients. Some vitamins, such as vitamin C, vitamin E, and carotenoids, have strong antioxidant and neuroprotective effects and are commonly found in plants.

Vitamin C, also known as ascorbic acid, has four hydroxyl functional groups, two of which are enol hydroxyl groups. Therefore, vitamin C is easily oxidized and dehydrogenated, which makes it extremely reductive and therefore also a highly effective antioxidant. The vitamin C transporter SVCT2 (Berger et al., 2003) can be expressed in both neurons and glial cells, and can regenerate the myelin of neuronal cells (Rohr et al., 2017). The regenerative function of vitamin C can further produce glial cells (Guo et al., 2018) and cells of the sciatic nerve (Li et al., 2019), but there is no evidence yet that such an effect extends to neurons. Vitamins have excellent neuroprotective effects against the oxidative stress induced by metal and non-metals, and can protect against lead-induced neuronal apoptosis in rats (Ebrahimzadeh-Bideskan et al., 2016), aluminum-induced neuronal apoptosis in Nile perch (Khalil and Hussein, 2015), iron-induced oxidative stress in rat brain tissue (Ganie et al., 2014), and arsenic-related neurological damage in rats (Sarkozi et al., 2016). Specifically, the ability of vitamin C to scavenge free radicals can reduce the degree of oxidative stress and increase the viability of nerve cells in the cerebral cortex and striatum (Ballaz and Rebec, 2019).

Vitamin E mainly differs from vitamin C in the sense that it is fat-soluble. Vitamin E features hydroxyl hydrogen on a diacetyl alcohol ring, which produces a strong reduction effect on oxygen free radicals and effectively inhibits lipid peroxidation. Therefore, vitamin E can reduce oxidative stress and the production of free radicals and prevent cognitive decline caused by aging. In an accelerated aging rat model, vitamin E supplementation reduced the number of nerve cells lost due to aging (La Fata et al., 2017), and improved memory and cognitive decline due to the loss of prefrontal cortical cells (Rafati et al., 2017). Vitamin E also increased the neuronal cell area of prefrontal cortical cells, the number of glial cells and neurons, the length of synapses, and plasticity effects (Rafati et al., 2018). It also prevented changes in the shape of dendrites of nerve cells that lead to learning deficits (Veinbergs et al., 2000) and increased the density of neuroreceptors in the hippocampus of rats with neurological injury (Sayyahi et al., 2018). The concentration of vitamin E in the brain is negatively correlated with the concentration of Aβ in the brain (Morris et al., 2015) and can help reduce the accumulation of Aβ in the rat brain and increase the clearance of Aβ in the blood (Nishida et al., 2009). Vitamin E also prevents the metabolism of 12-LOX, a key mediator of glutamate-induced neurodegeneration, by preventing arachidonic acid from entering the catalytic site of 12-LOX and enabling vitamin E to effectively prevent neuronal degeneration (Khanna et al., 2003). Furthermore, vitamin E can interact with vitamin C and flavonoids to enhance the ability of antioxidants to scavenge free radicals (Kadoma et al., 2006).

Carotenoids are widely present in plant pigments, and their chemical structure is a polymer of 8 isoprene and an oxidized derivative thereof, which is a precursor of vitamin A. Since the chemical structure of carotenoids contains many conjugated double bonds, electrons can enrich the structure while maintaining high chemical stability and fighting free radicals. At the same time, carotenoids also exhibit obvious effects for inhibiting lipid peroxidation, which can effectively prevent brain aging. According to nutrition epidemiology research, the middle-aged population consuming a diet rich in carotenoids generally scores higher on neuropsychological tests than that consuming an ordinary diet (Kesse-Guyot et al., 2014). This concept is supported by the fact that carotenoids can inhibit the formation of Aβ in the AD brain (Obulesu et al., 2011) and improve the secretion of oxidative stress and pro-inflammatory mediators (Mohammadzadeh Honarvar et al., 2017).

In the past 10 years, natural carotenoids, such as astaxanthin (Hussein et al., 2006), crocin (Asdaq and Inamdar, 2010), and lycopene (Agarwal and Rao, 2000), have been found to exhibit neuroprotective effects. Astaxanthin is the most potent antioxidant in flavonoids and is found in the leaves and fruits of plants. When supplementing with astaxanthin in the hippocampus, neonatal mice were found to show enhanced cognitive ability compared to adult mice (Yook et al., 2016). In terms of mechanism, astaxanthin can cross the blood–brain barrier (Petri and Lundebye, 2007) to exert its antioxidant (Pan et al., 2017), anti-inflammatory (Zhang et al., 2017b), and neuroplastic effects on the brain. Astaxanthin also attenuates the increase in CHOP and ER chaperone protein via the MAPK pathway and inhibits the influx of calcium ions (Grimmig et al., 2019) and upregulates HO-1 via the ERK1/2 pathway to protect against the neurotoxicity of Aβ (Lin et al., 2017). It is generally known that Aβ causes long-term dysfunction in the hippocampus of mice; however, the injection of crocin can improve the physiological state of cells in the hippocampus and the overall memory of mice (Lin et al., 2017). In experiments examining oxidative stress and inflammation, crocinin was shown to protect cells against neurodegeneration by activating the Akt/GSK, CREB/BDNF, and NF-κB signaling pathways (Zhang et al., 2018; Motaghinejad et al., 2019).

Finally, lycopene is widely present in tomatoes and has high neurotrophic value (Shi and Le Maguer, 2000). In a model of Aβ-induced neuronal injury, lycopene did not activate NF-κB, p65, or TLR4 expression (Tripathi et al., 2018), but restored mitochondrial morphological membrane potential and ATP levels (Qu et al., 2016) and inhibited Bax and mitochondria. The associated decrease in the level of Bcl-2 (Feng et al., 2016) created protective effects against inflammation and oxidative stress in nerve cells. In fact, after 14 days of eating tomatoes, the expression of dopaminergic neurons in the substantia nigra and striatum of small mammals was not reduced even under increased oxidative stress (di Matteo et al., 2009).

### Alkaloids

Alkaloids are strong antioxidants with a complex structure; the more nitrogen atoms that are exposed in the heterocyclic structure, the easier it is to combine with reactive oxygen and free radicals. There are many naturally occurring plant-derived alkaloids that have long been used to develop useful pharmaceutical substances (Khadem and Marles, 2011). The astragalus alkaloid and its derivatives are an important target for biological research because they are commonly found in food sources such as coffee, tea, and potatoes (Monteiro et al., 2016). The main derivatives of astragalus are methylxanthine, theophylline (1,3-dimethylxanthine), theobromine (3,7-dimethylxanthine), and caffeine (1,3,7-trimethylxanthine) (Li et al., 2017). Caffeine is considered to be a more beneficial derivative of astragalus to humans (Machnik et al., 2017) and can excite the central nervous system. Therefore, proper caffeine intake can be very beneficial to the nervous system in general (Cappelletti et al., 2015). Caffeine is an antagonist of adenosine A(1) and A(2A) receptors, which are closely related to the regulation of synaptic plasticity and energy metabolism in neurons (Cellai et al., 2018). This is particularly important for neurodegenerative diseases such as epilepsy (Masino et al., 2014), in which changes in the level of adenosine can be quite prevalent. Additionally, memory loss caused by chronic stress is linked to the A2AR receptor and caffeine acts as an antagonist to prevent and treat such symptoms (Kaster et al., 2015). Additionally, when considering cognitive decline caused by aging, dietary intake of caffeine can protect the brain by regulating the Bax/Bcl2 ratio, caspase-3, and PARP-1 levels to reduce oxidative stress, and COX-2, NOS-2, TNFα, and IL-1β to further reduce D-galactose-induced neuroinflammation and neurodegeneration (Ullah et al., 2015).

Another common alkaloid that affects the nervous system is the nicotine commonly found in tobacco. A large amount of smoking and nicotine intake is an important risk factor for death. By controlling the intake of nicotine, this recognized “killer” can become a “doctor.” The level of nicotine acetylcholine in the brain of smokers has been found to be much higher than in that of non-smokers and can promote the release of dopamine in the striatum (Jasinska et al., 2014). Nicotine acetylcholine receptors (nACHRs) (Hogg et al., 2003) can control the resting potential and excitatory conduction in neurons. In the AD brain, the α7 nicotinic acetylcholine receptor (α7nAChR) can be damaged with Aβ knots to form a complex that disrupts synaptic function (Ni et al., 2013) and using an antagonist of α7nAChR can actually inhibit the proliferation of microglia and reduce the expression of inflammatory factors IL-1β and TNF-α (Guan et al., 2015). Furthermore, it has been found in humans and animals that nicotine enhances the learning ability (Poorthuis et al., 2009), improves neuronal plasticity in the hippocampus (Alkadhi, 2018), treats depression (Motaghinejad et al., 2016), and can treat excitotoxicity at low concentrations (Sieber, 2012). Through the ERK1/2 pathway (Ju et al., 2017), the PI3K–AKT pathway (Huang et al., 2012), and the mitochondrial apoptotic pathway (Akaike et al., 2010), nicotine can also protect neurons from oxidative stress-induced apoptosis.

### Polysaccharides

A polysaccharide refers to the natural substance formed by polymerizing more than 10 monosaccharide molecules. Many polysaccharides have a scavenging effect on reactive oxygen species and free radicals and are responsible for many basic functions of the brain. They can bind to proteins and lipids, stabilize the structure of nerve cells and synapses (Martin, 2002), and are used as a source of cellular energy (Falkowska et al., 2015) and as a neurotransmitter precursor substance (Varki, 2017), which are essential nutrients for the brain (Nelson et al., 2012). Polysaccharides can exert many functions through the metabolism of the brain and can contribute to the formation of neurites and synapses in neurons (Miyata and Kitagawa, 2017). They can also provide energy substances such as lactic acid to neurons through the metabolism of glial cells (Falkowska et al., 2015) and in the AD brain can reduce the production of Aβ to reduce toxicity (Timmer et al., 2010), inhibit certain inflammatory factors, and increase resistance to oxidative stress (Jia et al., 2015). Polysaccharides are also known to exert neuroprotective effects on mitochondria by reducing the ratio of Bax/Bcl-2, caspase-3 (Yu et al., 2017), P-AKT, and phosphoric acid. Similar protective effects can be observed across numerous types of nerve cells by inhibiting GSK-3β (Hu et al., 2016) and activating ERK (Zhang et al., 2017d) to reverse oxidative damage.

### Active Peptides

A biologically active peptide, known as a polypeptide, can be obtained via the process of protein hydrolysis. The function of a polypeptide is related to its amino acid composition and structure. When ingested by humans, plant proteins are hydrolyzed by the digestive system and many small peptides and amino acids are obtained. These amino acids often activate PKA while glutamate transporters of neurons and astrocytes can promote the cascade of the MAPK reaction (Steyfkens et al., 2018). There are a wide variety of active peptides that include antioxidant peptides and opioid peptides that have important neuroprotective effects. Glutathione is an essential peptide in plant growth and development and plays an important role in the antioxidant system of animals. Specifically, glutathione peroxidase can remove large numbers of free radicals for antioxidant metabolism (Noctor et al., 2012). The mitochondrial metabolism of reactive oxygen species also requires the use of glutathione (Fernandez-Checa et al., 1998). These processes are important to counteract oxidative stress (Dukhande et al., 2013) by causing an increase in Bcl-2. Recently, it has been confirmed that in the ischemia–reperfusion model in rodents, cerebral nerve cell damage is associated with glutathione depletion, and post-ischemia recovery is associated with an increase in the concentration of glutathione (Won et al., 2015). By detecting the concentration of glutathione in the brain of AD patients, it was found that the concentration of glutathione was less than that of normal brains and showed an age preference (Rae and Williams, 2017). Furthermore, peptides of soy protein, rice protein, and hydrolysate of wheat gliadin derived from wheat (Teschemacher, 2003) have opioid activity and can cause an increase in the antioxidant and methylation capacity of nerve cells (Trivedi et al., 2014) and lower the NO level across the cellular environment (Yin et al., 2015). Opioid peptides act in conjunction with opioid receptors, the activation of which can inhibit P38 phosphorylation via PCK and MAPK activation, prevent cell apoptosis, and scavenge free radicals to protect nerve cells (Staples et al., 2013). In addition, scientists have recently extracted Rubisco peptides from spinach leaves that have been shown to have significant anti-anxiety effects (Kimura et al., 2018).

## Plant Antioxidants Participate in the Coordinated Protection of the Nervous System

The nervous system is integrated with the entire human body, and plant-derived antioxidant substances are absorbed by the digestive system and then circulated throughout the body. Therefore, nerve cells in the whole body may be protected. In the nervous system, some nerve cells regulated the immune response and cell repair. Plant-derived antioxidants can protect this part of the nervous system with immune function, thereby increasing its ability to resist damage and repair itself.

### The Protective Effect of Glial Cells on Neurons

Two of the most prevalent cell types in the brain are neurons and glial cells. The primary function of neurons and glial cells is to transmit information and to provide nutrients, respectively. Through co-culture experiments with glial cells and neurons, it was found that glial cells can help neurons resist oxidative stress by secreting antioxidants or helping antioxidant precursors. Glutathione has important functions in the antioxidant metabolism of the brain, but different cells in the brain have different requirements for glutathione precursors, which provide a defense against oxidation (Dringen, 2000). Plant nutrients eventually enter the bloodstream through dietary digestion, but due to the presence of the blood–brain barrier, substances in the blood are not fully absorbed and utilized by the brain. The neuro-glio-vascular unit, which is mainly composed of glial cells, is formed around the blood vessels of the brain; however, connexin (Cx) channels allow nutrients to enter the brain and leave harmful substances in the blood vessels. The pathological state of glial cells regulates the blood–brain barrier (De Bock et al., 2017) and therefore these cells can be thought of as guardians that protect neurons against oxidative stress (Liu et al., 2018) via the Saposin C/GPR37L1/GPR37 pathway. When the brain is exposed to a certain degree of oxidative stress, glial cells can cause neuronal damage, but also secrete a large amount of neuropeptides to protect both glial cells and neurons, and improve the brain’s defense against oxidative stress (Ghouili et al., 2018). Overall, glial cells can play an important role in the recovery of brain function (Toledano et al., 2016), so the protection of glial cells can be considered directly related to nervous system protection.

### Protective Effect of Microglia on Neurons

**FIGURE 2:**
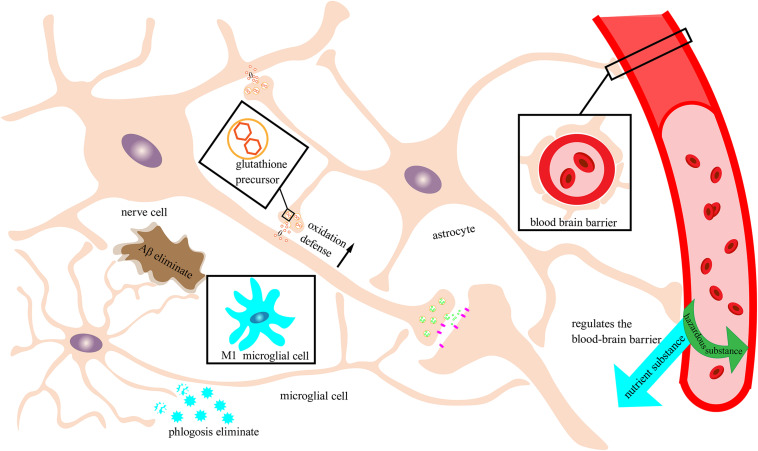
Neuronal protection by the immune nervous system. Schematic of an immune-functioning nervous system that is protecting neurons through the combined action of astrocytes and microglia. Astrocytes tightly combine with the blood vessels of the brain to form a blood–brain barrier to filter out harmful substances and allow beneficial nutrients to enter the brain. Moreover, astrocytes can secrete glutathione precursors to strengthen the oxidative defense of neurons. Microglia in this example are depicted as exhibiting the M2 phenotype with immune function rather than the M1 phenotype that can trigger neuroinflammation. M2 microglia can recognize and break down Aβ and inflammatory factors and reduce damage to neurons.

Microglia are monocytes that can enter the central nervous system and generally exhibit either the M1 or M2 phenotype. Microglia with the M1 phenotype can show signs of inflammation under specific environments whereas those with the M2 phenotype generally do not. Microglia have a unique immune function in the brain (Tang and Le, 2016) by activating synaptic plasticity. Microglia also help remove brain toxins and cellular metabolic waste to maintain the homeostasis of the brain (Chen and Trapp, 2016). In a study of aging mice, neuronal degeneration was clearly seen as a function of aging (Hasegawa-Ishii et al., 2011) and the degradation and morphological changes of microglial cells increased the risk of brain damage. Nevertheless, through the timely treatment with polyphenols, the brain’s ability to resist neurodegenerative diseases significantly improved (Conde and Streit, 2006; Bickford et al., 2017). Microglial cells can also be activated to clear the accumulated Aβ in the brain (Lee and Landreth, 2010), while resveratrol (Cai et al., 2017), sinomenine (Feng and Zhang, 2019), and ganoderma polysaccharide (Shukla and Sharma, 2011) can induce protection against Aβ damage. Recently, it was also found that plant extracts can regulate microglia to express the M2 phenotype more than the M1 phenotype (Yang et al., 2017) to reduce neuroinflammation and nerve cell damage and to clear Aβ (Figure 2). Additionally, oral theaflavin can improve the cognitive behavior of LPS-induced neuroinflammation by inhibiting the activation of M1-type microglia (Ano et al., 2019). Curcumin can also protect BV-2 microglia by effectively reducing the index of oxidative stress in glaucoma mice (Yue et al., 2014). Furthermore, flavonoids in plants have the ability to regulate microglial activation (inhibiting M1-type activation), which allows flavonoids to reduce various inflammatory factors in the brain (Spagnuolo et al., 2018).

### Protection Against the Effects of Nervous System Aging on Degenerative Diseases

Aging is the biggest cause of AD, mainly manifesting as the accumulation of Aβ cell tangles, which eventually lead to memory (Crowther and Goedert, 2000; Selkoe and Hardy, 2016) and cognitive decline (De-Paula et al., 2012). Microglia play important roles in the immune regulation in the brain, monitoring CNS and inflammatory factors, and clearing Aβ and cell debris (Sarlus and Heneka, 2017). Antioxidants such as glutathione and vitamin C cooperate with microglia to respond to acute and chronic oxidative stress (Freitas et al., 2017). The lack of vitamin B affects the health of the entire nervous system and the gradual degradation of neurons can lead to PD, AD, or amyotrophic lateral sclerosis. Vitamin B deficiency can affect the health of the entire nervous system; AD and PD are caused by the gradual degeneration of nerve cells in the brain and amyotrophic lateral sclerosis (Freitas et al., 2017). The specific combination of folic acid and vitamins B, C, and E as well as others has improved the plasticity of nerve cell synaptic dysfunction (Kihara and Shimohama, 2004) and has restored the cognitive ability and memory in AD patients in various clinical trials (van Wijk et al., 2014). Furthermore, neurotrophic and antioxidant substances in plants have anti-aging effects on the nervous system and promote the functional integrity of nerve cells. On one hand, nutrients and antioxidants can promote and activate immune cells in the nervous system, which can provide antioxidant and anti-inflammatory effects and nutrient supply to protect the health of the brain and the integrity of CNS function. On the other hand, antioxidant substances in plant cells can enter the brain and the whole body through the blood and can play an important role in the enhancement of the antioxidant defense of the whole nervous system.

The functional integrity of the nervous system is inseparable from the cooperation of the nerve cells. Nerve cells each carry out their normal functions and ensure the reasonable operation of the nervous system. In terms of anti-oxidation, anti-aging, and neuroprotection, plant-derived antioxidants can eliminate free radicals in cells of various parts of the nervous system to achieve neuroprotection. The elimination of free radicals can also activate the immune regulation mechanism in the nervous system to achieve neuroprotection. In the treatment and prevention of nerve-related diseases, plant-derived antioxidants can affect both nerve cells and the nervous system as a whole. In the process of research and development of plant antioxidants, the effects of nutrients on nerve cells and the nervous system should be more widely linked so that the nutrients in plants are fully developed and utilized.

## Author Contributions

XC: writing – original draft preparation and software. QL: supervision and data curation. YL: conceptualization, writing – reviewing and editing, and investigation. All authors contributed to the article and approved the submitted version.

## Conflict of Interest

The authors declare that the research was conducted in the absence of any commercial or financial relationships that could be construed as a potential conflict of interest.
